# Impact of hemoadsorption with CytoSorb® on meropenem and piperacillin exposure in critically ill patients in a post-CKRT setup: a single-center, retrospective data analysis

**DOI:** 10.1186/s40635-025-00716-0

**Published:** 2025-01-18

**Authors:** Golschan Asgarpur, Franz Weber, Peggy Kiessling, Nilufar Akbari, Fabian Stroben, Bernadette Kleikamp, Charlotte Kloft, Sascha Treskatsch, Stefan Angermair

**Affiliations:** 1 Freie Universität Berlin and Humboldt-Universität Zu Berlin, Department of Anesthesiology and Intensive Care Medicine, Charité—Universitätsmedizin Berlin, Campus Benjamin Franklin, Berlin, Germany; 2 Department of Clinical Pharmacy and Biochemistry, Institute of Pharmacy, Freie Universitaet Berlin, Kelchstr. 31, 12169 Berlin, Germany; 3 Graduate Research Training Program PharMetrX, Freie Universitaet Berlin/Universität Potsdam, 12169 Berlin, Germany; 4 Labor Berlin—Charité Vivantes GmbH, 13353 Berlin, Germany; 5 Freie Universität Berlin and Humboldt-Universität Zu Berlin, Institute of Biometry and Clinical Epidemiology, Charité—Universitätsmedizin Berlin, Charitéplatz 1, 10117 Berlin, Germany

**Keywords:** CytoSorb®, Extracorporeal therapy, Hemoadsorption, Therapeutic drug monitoring (TDM), Meropenem, Piperacillin/tazobactam

## Abstract

**Purpose:**

CytoSorb® (CS) adsorbent is a hemoadsorption filter for extracorporeal blood purification often integrated into continuous kidney replacement therapy (CKRT). It is primarily used in critically ill patients with sepsis and related conditions, including cytokine storms and systemic inflammatory responses. Up to now, there is no evidence nor recommendation for the use of CS filters in sepsis (22). There is limited clinical data on the effect of CS on the plasma concentrations of beta-lactams. We aimed to evaluate the statistical and clinical impact of CS in a post-filter CKRT-CS setting on the plasma concentrations of the antibiotics meropenem and piperacillin in critically ill patients.

**Methods:**

Patients admitted to the intensive care unit (ICU) who received a prolonged infusion of piperacillin or meropenem with CS-combined CKRT were included in this retrospective analysis. TDM (therapeutic drug monitoring) plasma blood samples were collected at three different points. The differences in antibiotic concentrations between Pre, Intra, and Post were statistically compared to evaluate the total and isolated contributions of CKRT and CS to antibiotic removal. CS, CKRT and combined clearance (CL) values were calculated. The hypothesis was that the CS filter would have no clinically relevant impact on antibiotic levels.

**Results:**

207 TDM samples were taken from 24 critically ill patients requiring beta-lactam antibiotics. Among these, 129 were meropenem samples, and 78 were piperacillin samples. A decrease in both antibiotic levels was observed between Pre and Intra, and Pre and Post, and the median relative difference between was >15% (meropenem: Pre–Intra 34.8%, Pre–Post 35.8%; piperacillin: Pre–Intra 41.1%, Pre–Post 34.7%), indicating a statistically and clinically significant effect of CKRT on both antibiotic exposures. No significant difference was observed between Intra and Post indicating no clinically relevant drug removal via the CS filter. Changes in CL attributed to CS were minimal, with combined CL differing by ≤8.60% compared to CKRT clearance.

**Conclusion:**

The application of CS does not appear to significantly affect plasma concentrations of meropenem and piperacillin in critically ill patients.

**Supplementary Information:**

The online version contains supplementary material available at 10.1186/s40635-025-00716-0.

## Introduction

Sepsis and septic shock are life-threatening conditions characterized by organ dysfunction resulting from a dysregulated inflammatory host response to severe infection [1]. Pro-inflammatory and anti-inflammatory cytokines play a crucial pathophysiological role in developing sepsis. A large variety of cells produce cytokines, which are important for host defence, wound healing and other essential host functions [2, 3]. While they are important for homeostatic processes, excessive cytokine release leads to widespread tissue injury, potentially resulting in organ dysfunction [4]. Thus, the therapeutic reduction of these cytokines is currently a significant area of research [5, 6].

Besides investigations on immunomodulatory therapies, hemoadsorption technologies such as CytoSorb® (CS) filters are available to remove cytokines in septic patients [7, 8].

As a hemoadsorption filter for extracorporeal blood purification, it is often integrated into continuous renal replacement therapy (CKRT). The CS adsorbent contains divinylbenzene copolymer beads sized 300–800 µm with a biocompatible coating. CS filter enables targeted adsorption of molecules sized between 6–70 kDa from the blood, effectively targeting most sepsis-relevant cytokines, but also hormones, anti-inflammatory mediators and clotting factors [9]. However, there is growing evidence suggesting that the use of CS may have negative effects on patient outcomes and the current evidence remains limited [1, 10]. While effectively attenuating cytokine concentrations during systemic inflammation, thus having the potential of being beneficial in conditions characterized by excessive cytokine release, a meta-analysis including 34 studies with 1297 patients did not show any positive effect of the CS adsorbent on mortality [11]. One possible reason could be the removal of antibiotics, which could lead to a reduction in effective doses. This, in turn, could result in ineffective antibiotic therapy for the underlying infection, deterioration of the patient’s clinical outcome, and potentially promote the development of antibiotic resistance. Available in vitro data suggest minimal removal of aminoglycosides and almost complete removal of glycopeptides (e.g., vancomycin and teicoplanin) [12]. However, there is limited clinical data on the effect of CS on the plasma concentrations of other commonly used antibiotics, such as meropenem and piperacillin (in combination with tazobactam). We analyzed the plasma levels of the beta-lactam antibiotics piperacillin and meropenem during CS-enriched CKRT in critically ill patients, simultaneously sampling from various points within the circuit to assess the potential impact of CS on antibiotic exposure. The hypothesis was that the CS filter does not have a clinically relevant impact on the removal of meropenem and piperacillin plasma concentrations and therefore does not necessitate additional dosing of either antibiotic during CS therapy.

## Material and methods

### Study design and patients

A single-center, retrospective data analysis was conducted at Charité—Universitätsmedizin Berlin, including patients aged 18 years or older, who were admitted to the intensive care unit (ICU) of the Department of Anesthesiology with Intensive Care Medicine, Campus Benjamin Franklin, between January 2020 and December 2023. The therapeutic drug monitoring (TDM) process described was initially implemented based on a standard operating procedure (SOP) established at our institution. This SOP was developed in response to frequent implausible measurements of piperacillin and meropenem levels observed in patients undergoing CKRT in combination with CytoSorb®. These inconsistencies raised questions about the influence of the CytoSorb® adsorber on drug concentrations.

During the clinical implementation of the SOP, we observed that contrary to prior assumptions, the CytoSorb® adsorber did not significantly impact the measured drug levels. Based on these findings, we decided to conduct a retrospective data analysis to investigate this phenomenon further. For this retrospective data analysis, ethical approval was obtained from the Charité ethics committee. The committee explicitly stated that no patient consent was required for this type of retrospective analysis, as the data were pre-existing, anonymized, and collected as part of routine care. This classification aligns with local ethical standards and regulations for retrospective studies.

The retrospective data analysis was approved by the ethics committee of the Charité—Universitätsmedizin Berlin (EA4/015/24) and registered in the German Clinical Trial Register (DRKS ID: 00034788). The study adhered to the principles outlined in the Declaration of Helsinki. To gather patient information, Structured Query Language (SQL) inquiries were executed using the hospital’s electronic medical record system (COPRA System GmbH, Sasbachwalden, Germany, and SAP AG, Walldorf, Germany).

Patients eligible for the analysis underwent veno-venous CKRT with a simultaneous CS filter via high-volume Sheldon’s catheter insertion according to the department’s standard operating procedure (SOP) and international recommendations. Further inclusion criteria were an infection with an indication for anti-infective therapy with meropenem or piperacillin. The dosing regime for carbapenems is based on international guidelines and clinical standards [6–8]. Both antibiotics were administered as prolonged intravenous infusions: piperacillin for 3 or 4 h, and meropenem exclusively for 4 h. Pregnant or potentially pregnant individuals were excluded from the study.

**Fig. 1:**
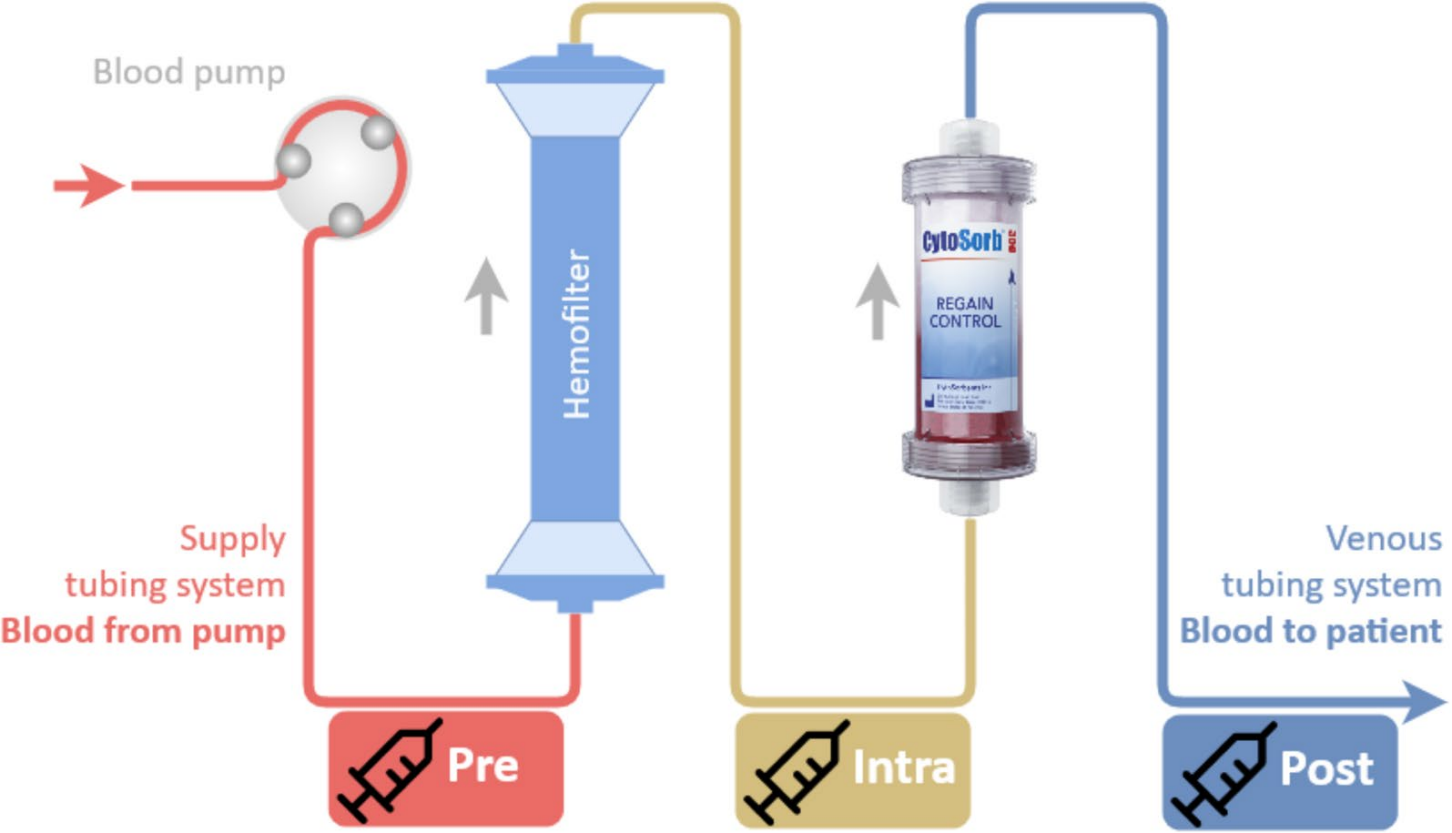
Schematic representation of the CKRT-CS set-up and the corresponding blood sampling sites Pre, Intra, and Post for meropenem and piperacillin quantification. *CKRT* renal replacement therapy, *CKRT-CS* continuous renal replacement therapy followed by CS, *Pre* before CKRT-CS setup, *Intra* between CKRT and CS, *Post* after CKRT-CS setup. Image adapted with courtesy of CytoSorb® Europe, Berlin, Germany

All patients received CKRT with the CS 300 mL hemoadsorber (CSents Corporation, Princeton, New Jersey, USA) placed in a post-filter position within the CKRT circuit according to clinical standards (Fig. 1). Before use, the CKRT and CS filters were primed with 0.9% sodium chloride solution according to the manufacturer’s instructions. The CS filter was replaced every 18–24 h. The CKRT was performed using the multiFiltratePRO Ci-Ca system (Fresenius Medical Care GmbH, Bad Homburg, Germany) for pre-dilution continuous veno-venous hemodialysis with the Ultraflux AV 600 polysulfone capillary hemofilter (Fresenius Medical Care, Bad Homburg, Germany). Flow rates through the hemoadsorption device were maintained at 200 mL/min. No hemofilter change occurred within the last 24 h before TDM.

TDM was conducted simultaneously at three measurement points (“triplet”): before the hemofilter (Pre), between the hemofilter and the CS filter (Intra), and after the CS filter (Post) (Fig. 1). All blood samples were collected at the end of the dosing interval (minimum drug concentration). TDM samples were always taken 24 h after the start of CKRT with a CS filter at all three sampling locations.

### Chromatography and mass spectrometry

Immediately after collection, samples were sent to Labor Berlin (Labor Berlin—Charité Vivantes GmbH, Berlin), centrifuged and stored at −20 °C until antibiotic concentrations were determined by using high-performance liquid chromatography coupled with a tandem mass spectrometer. Both analytes were successfully validated in a calibration range of 2.0–115 µg/mL for meropenem, and 2.0–100 µg/mL for piperacillin by the GTFCh guidelines [14]. Concentrations outside the calibration range were diluted and re-evaluated [10, 11].

To assess whether the administered antibiotics are adsorbed onto the CS filter the following assumption was made: adsorption on the CS filter can only be detected if the deviation of the individual measured values for meropenem and piperacillin between Intra and Post is greater than the respective estimated measurement uncertainty for these parameters. The estimation of the measurement uncertainty was carried out by ISO 21748. The results of the last five interlaboratory test samples were compared. The measurement uncertainty for meropenem was 13.9% and for piperacillin 5.4% regarding 5 external quality controls and 10 precision measurements.

### Statistical analysis

The primary endpoint was the change in anti-infective plasma levels measured as the median relative difference (Sup. Eq. S5-S7) between the sampling locations with bootstrapped 95% confidence intervals (CI). A power analysis was conducted in advance using the software G*Power (v. 3.1.9.7). Fifteen patients have a power of 80% to detect an effect size (dz) of 0.80 with a Wilcoxon signed-rank test for paired samples and a two-sided significance level of 0.05. Nine can detect an effect size (dz) of 1.10 with a Wilcoxon signed-rank test for paired samples under the same power and significance level.

The Wilcoxon signed-rank test with a two-sided significance level of 0.05 for within-group comparison was used to compare the median antibiotic concentrations between two sampling locations. Box–whisker and violin plots were created to visualize the concentration differences within a triplet between sampling locations. Adjustment for multiple testing was deliberately omitted. The comparisons included Pre vs. Post, Pre vs. Intra and Post vs. Intra, each for meropenem and piperacillin. Determined p-values were exploratory and did not allow for confirmatory generalization. Additionally, a Wilcoxon signed-rank test with average Pre-, Intra- and Post-values per patient was conducted. Clearance (CL) values based on the documented blood flow rates (*Qb*) and the median concentration values at Pre ($${C}_{Pre})$$, Intra ($${C}_{Intra})$$, and Post ($${C}_{Post})$$ were calculated [15] for the CL of CKRT ($${CL}_{dial}$$), CS ($${CL}_{cyto}$$), and CKRT and CS combined ($${CL}_{comb}$$) (Eq. S1–S3). Additionally, relative differences between $${CL}_{dial}$$ and $${CL}_{comb}$$ were determined to quantify the impact of CS on meropenem and piperacillin $${CL}_{comb}$$ (Eq. S4).

A deviation in concentrations of more than 15% was defined as the limit above which a clinically relevant influence on meropenem and piperacillin exposure could be expected taking the imprecision of the bioanalytical method into account [16]. The calculation of median relative differences (Eq. S5–S7), the bootstrap analysis and the generation of all plots were performed in R (v.4.2.1; R Core Team, 2022; https://www.R-project.org/).

## Results

207 TDM samples were taken at Pre, Intra, and Post (=69 triplets) from 24 critically ill patients requiring beta-lactam antibiotics for infection treatment. Among these, 129 were meropenem samples (43 triplets; Sup.Fig. S2 from 15 patients), and 78 were piperacillin samples (26 triplets; Sup. Fig S4 from 9 patients). There were no differences between the antibiotic treatment groups in terms of gender, age, weight, or SOFA score at the time of inclusion in the study. Additionally, other factors influencing pharmacodynamics, such as albumin and creatinine levels, showed no differences between the groups (Table 1). Dialysis setting parameters showed no differences either, or the ultrafiltration rate was adjusted daily according to the cardio-circulatory situation, which is not known to alter the adsorption rate of agents. All patients required treatment with beta-lactam antibiotics. 15 patients received meropenem, and 9 patients received piperacillin. Both antibiotics were administered as prolonged intravenous infusions: piperacillin for 3 h (44.4% of cases) or 4 h (55.5% of cases), and meropenem exclusively for 4 h. The single dose of meropenem was 1 g (range: 1–2 g), and piperacillin was administered as 4 g in fixed combination with 0.5 g tazobactam.

**Table 1:** Baseline characteristics, laboratory measurements, dialysis settings, and antibiotic therapeutic drug monitoring (TDM) samples for patients treated with meropenem or piperacillin

Characteristics	Frequency (%) or median (IQR)	
	Meropenem	Piperacillin
*Patients*		
No. of patients	15 (–)	9 (–)
No. of female patients	6 (40)	5 (55.5)
Total body weight [kg]	80 (76.5–85)	71.5 (68.8–75.5)
Body height [cm]	172 (165–178)	168 (161–172)
Age [years]	70 (61.5–74)	77 (66–78)
SOFA score	13 (8–15)	11.5 (9.50–13.5)
*Laboratory measurements*		
Creatinine [mg/dL]	1.18 (0.78–1.74)	2.12 (1.35–2.62)
IL-6 [pg/mL]	354 (110–433)	–
PCT [ng/mL]	1.95 (0.73–6.27)	–
CRP [mg/L]	195 (118–269)	243 (166–313)
Ferritin [ng/mL]	1219 (1050–2024)	–
LDH [U/L]	463 (360–579)	989 (813–1010)
Urea [mg/dL]	46.0 (32.0–71.0)	61.0 (42.0–86.0)
Albumin [g/dL]	20.6 (17.8–22.7)	19.8 (18.9–23.7)
*Dialysis setting*		
Blood flow [mL/min]	100 (100–100)	100 (100–100)
Dialysate flow [mL/min]	2000 (2000–100)	2000 (2000–100)
Ultrafiltration rate [mL/h]	810 (0–1815)	385 (0–1045)
*Antibiotic TDM samples*		
No. of samples Pre CKRT-CS setup	43 (–)	26 (–)
No. of samples Intra CKRT-CS setup	43 (–)	26 (–)
No. of samples Post CKRT-CS setup	43 (–)	26 (–)
Number of triplet samples (Pre, Intra, Post) per patient	2 (1–4.5)	3 (2–3)
Concentration Pre CKRT-CS setup [mg/L]	33.3 (25.4–46.0)	101 (44.8–120)
Concentrations Intra CKRT-CS setup [mg/L]	20.2 (15.2–29.8)	43.4 (27.7–68.0)
Concentrations Post CKRT-CS setup [mg/L]	20.1 (16.5–30.0)	48.0 (28.6–77.0)

Data are presented as frequency (%) for categorical and median (IQR) for continuous variables***.**** SOFA* Sequential Organ Failure Assessment, *IL-6* interleukin-6, *PCT* procalcitonin, *CRP* C-reactive protein, *LDH* Lactate dehydrogenase, *TDM* therapeutic drug monitoring, *CKRT-CS* continuous renal replacement therapy followed by CS, *Pre* before CKRT-CS setup, *Intra* between CKRT and CS, *Post* after CKRT-CS setup

**Fig. 2:**
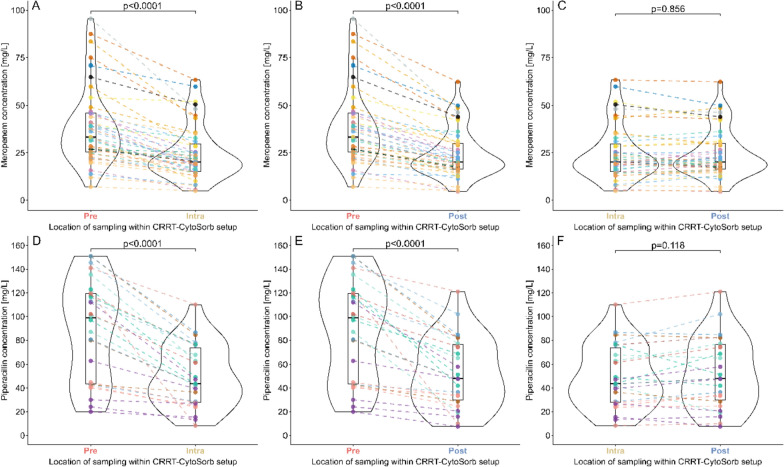
Comparison of meropenem (top) and piperacillin (bottom) concentrations at different sampling locations within the CKRT-CS setup (Pre, Intra, Post): (A and D) concentrations at Pre and Intra, (B and E) concentrations at Pre and Post, (C and F) concentrations at Intra and Post. All paired samples were taken at the same time within one patient’s CKRT-CS setup. Dashed lines connect concentrations within the same patient at the same sampling time point. Colors of dots and dashed lines: individual patients. Box–whisker plots: median, interquartile range (IQR), and whiskers smallest and largest values within 1.5 * IQR from the first and third quartiles. Integrated violin plots: shape of concentration distribution. *CKRT-CS setup* continuous renal replacement therapy followed by CS, *Pre* before CKRT-CS setup, *Intra* between CKRT and CS, *Post* after CKRT-CS setup

207 TDM samples were taken at Pre, Intra, and Post (=69 triplets) from 24 critically ill patients requiring beta-lactam antibiotics for infection treatment. Among these, 129 were meropenem samples (43 triplets; Sup.), and 78 were piperacillin samples (26 triplets; Sup.). Meropenem and piperacillin differences within triplets were detected between Pre- and Intra-measurements and between Pre- and Post-measurements (Table 2). No differences were observed between Intra- and Post-measurements for either drug (Fig. 2; Sup. Fig. S1–S6). For both antibiotics, significant differences were detected between meropenem and piperacillin concentrations only for Pre vs Intra (*p* < 0.0001 for meropenem, *p* < 0.0001 for piperacillin) and Pre vs Post (*p* < 0.0001 for meropenem, *p* < 0.0001 for piperacillin), while no differences were detected between Intra and Post (*p* = 0.856 for meropenem, *p* = 0.118 for piperacillin; Sup. Table S1). An equivalent result was obtained from the Wilcoxon signed-rank test with average Pre-, Intra- and Post-values per patient (Sup. Table S2).

**Table 2:** The median relative differences with bootstrapped 95% confidence intervals and the median decrease with interquartile range between sampling locations for meropenem and piperacillin within the CKRT-CS setup (Pre, Intra, Post)

Antibiotic	Comparison	Median relative difference (%)	95% confidence interval (%)	Median decrease (mg/L)	IQR (mg/L)
Meropenem	Pre vs. Post	34.8	30.0–42.0	12.2	12.7
	Pre vs. Intra	35.8	26.0–39.5	11.9	12.2
	Intra vs. Post	8.63	5.63–10.7	2.95	3.15
Piperacillin	Pre vs. Intra	41.1	36.5–46.7	37.1	44.5
	Pre vs. Post	34.7	26.2–39.9	31.3	42.8
	Intra vs. Post	10.7	4.87–15.6	7.55	6.35

*CKRT-CS setup* continuous renal replacement therapy followed by CS, *Pre* before CKRT-CS setup, *Intra* between CKRT and CS, *Post* after CKRT-CS setup

For meropenem, the ${CL}_{dial,mero}$ was 39.3 mL/min, while the ${CL}_{cyto,mero}$ was 0.50 mL/min and the ${CL}_{comb,mero}$ 39.6 mL/min. The ${CL}_{dial,mero}$ was 0.76% lower (−0.30 mL/min) compared to the ${CL}_{comb,mero}$. For piperacillin, the ${CL}_{dial,pip}$ was 57.0 mL/min, while the ${CL}_{cyto,pip}$ was -10.6 mL/min and the ${CL}_{comb,pip}$ 52.5 mL/min. The ${CL}_{dial,pip}$ was 8.60% (4.55 mL/min) higher compared to the ${CL}_{comb,pip}$. For meropenem and for piperacillin changes in CL attributed to CS did not surpass the threshold of 15%.

## Discussion

This retrospective data analysis demonstrates no significant adsorption of meropenem and piperacillin by the CytoSorb filter at the time of therapeutic drug monitoring. By comparing Pre-, Intra-, and Post-antibiotic concentrations from 207 real-world TDM samples, we confirmed that the CS filter does not remove meropenem and piperacillin. Thus, based on our data, additional dosing may not be required when CS filters are used in the CKRT-circuit. Additionally, our study corroborates previous findings of a statistically significant removal of these antibiotics by CKRT, highlighting the need for close monitoring and potential dosing adjustments [17].

For piperacillin, slight increases in concentrations between Intra and Post were observed. Furthermore, a negative ${CL}_{cyto,pip}$ and a decrease of ${CL}_{comb,pip}$ compared to ${CL}_{dial,pip}$ were calculated. The deviating observation compared with meropenem might be explained by measurement variability but might have also been caused by possible redistribution effects, previously described primarily for more lipophilic beta-lactam antibiotics [12]. Still, the CS effects on meropenem and piperacillin concentrations and extracorporeal CL were below the clinical threshold of 15% and are unlikely to impede successful antibiotic therapy. CKRT represented the predominant cause of changes in concentrations and ${CL}_{comb}$ for both antibiotics.

Animal studies investigating the effects of the CS filter on whole-body clearance and resulting theoretical dosing recommendations for antimicrobial agents suggest that no significant dosage adjustment is necessary for meropenem and piperacillin [5, 13]. Conversely, other antibiotics, such as vancomycin and linezolid, require additional dosing during CS treatment [17, 18]. We only investigated beta-lactams, whereas there appears to be a significant adsorption effect of CytoSorb® filters on fluoroquinolones [19]. However, precise pharmacokinetic data for in vivo antibiotic removal during hemoadsorption remains scarce, and a case report previously even suggested that meropenem was filtered out in a severely ill patient [20]. Overall, profound clinical data regarding the removal of meropenem and piperacillin by a cytokine filter in humans are insufficient. Furthermore, little is known about the intraindividual differences in antibiotic concentrations within the CKRT-CS setup of individual patients, which we addressed by measuring not only plasma but Pre-, Intra- and Post-concentrations of both antibiotics.

König et al. showed in an in vitro experiment that antibiotics are absorbed onto the surface of the CS hemoadsorber and are removed by the combined CKRT system to varying but clinically relevant degrees. The adsorber surface of the CS filter appears to become saturated, leading to a decrease in removal over time [21]. However, in vitro conditions cannot always be translated to in vivo conditions due to the effects of volume and distribution, protein binding, and competition with numerous other substances.

In vivo studies showed a complex influence of renal function and CKRT on antibiotic plasma levels [22]. Our results suggest that the hemofilter, as opposed to the CS absorber, is responsible for reducing antibiotic levels in a CKRT-CS setup.

Determining the optimal dosing regimen of antibiotics in critically ill patients is challenging due to numerous variables affecting the drugs’ pharmacokinetics (PK) and pharmacodynamics (PD). Factors such as alterations in the volume of distribution (*V*_*d*_), protein binding, and body clearance, often caused by impaired perfusion and organ dysfunction or failure, contribute to this complexity [23]. This challenge is further confounded in patients undergoing CKRT in combination with CS [12]. Inadequate dosing due to adsorption to the CS filter might lead to sub-therapeutic antibiotic levels, which can contribute to therapeutic failure and the development of bacterial resistance [24]. In critical care settings, particularly in septic patients or those with multidrug-resistant infections, the balance between removing inflammatory mediators and preserving effective antibiotic levels is crucial.

In patients undergoing CKRT, significant variability in antibiotic pharmacokinetics complicates empirical dosing, underlining the need for therapeutic drug monitoring. In our study, the dialysis settings for blood flow and dialysate flow were kept constant, and the patient characteristics and disease severity were comparable across the cohort. While determining outcomes was not the primary aim of the study, it is an intriguing question that warrants further investigation. Additional research is needed to explore the observed relative decrease in clearance at higher CKRT effluent rates [25].

However, clinicians should monitor antibiotic levels and adjust dosing regimens to ensure effective treatment. In critically ill patients, it is already challenging to achieve adequate antibiotic dosing due to fluctuating distribution volumes. The addition of an extracorporeal procedure with an integrated filter further complicates this task. According to our data, adjusting antibiotic dosing when using a CS filter is not necessary, even after a filter exchange. Ongoing research is essential to optimize their use in clinical practice and to develop guidelines that balance antibiotic therapy with blood purification.

## Limitation

One of the major limitations is the small number of patients enrolled as well as the retrospective character of the study. Some patients were included with only one sample, others with more samples, which is transparently shown in the violin plots via dots and colors, but is a limitation of this study.

Furthermore, the timing of antibiotic sampling during CytoSorb therapy was unsuitable to capture potential time-dependent adsorption effects of CS, previously described in literature [12]. More frequent sampling throughout CS therapy would be required to investigate these phenomena comprehensively. However, the primary focus of our study was to evaluate the clinically relevant impact of CytoSorb in a real-world setting. Our primary focus was on analyzing drug concentrations in plasma through therapeutic drug monitoring (TDM). While effluent measurements could provide valuable insights into drug clearance via the CKRT circuit, the retrospective nature of our study and the data available precluded such measurements. Acknowledging the potential value of investigating stand-alone cytokine adsorption in future studies, at our institution, we do not offer CytoSorb® therapy as a stand-alone treatment. According to our SOPs, CytoSorb® filters are exclusively used in combination with CKRT. This approach reflects our institutional protocol, which aims to integrate CytoSorb® therapy into existing CKRT workflows for critically ill patients, ensuring both consistency in practice and optimized resource utilization. As a result, we currently do not have data on stand-alone CytoSorb® therapy in our patient cohort.

Yet, to the best of our knowledge, this is the first study that compared interindividual meropenem and piperacillin concentrations at Pre-, Intra- and Post-sampling locations within a CKRT-CS setup in human patients, thereby strengthening the evidence of our findings. Furthermore, no generalization for other beta-lactam antibiotics is possible based on our results. Other compounds need to be assessed individually to understand the concentration levels in CKRT with and without a CS filter.

## Conclusion

The adsorption of antibiotics by CS filters is a complex process influenced by multiple factors. The effect of CS filters on antibiotic therapy necessitates careful consideration. Our study did not identify significant adsorption and thus removal of meropenem and piperacillin by the CS filter, indicating that there is no need to adjust the dosing of these two important antibiotics in critically ill patients. However, clinicians must monitor antibiotic levels in these patients during CKRT to ensure effective treatment.

## Take home message

Meropenem and piperacillin plasma levels are not reduced by CytoSorb filter.

## Supplementary Information

Additional file 1.

## Data Availability

The datasets used and/or analyzed during the current study are available from the corresponding author on reasonable request.

## Abbreviations

C: Celsius
CI: Confidence intervals
CKRT: Continuous renal replacement therapy
CKRT-CS: Continuous renal replacement therapy followed by CytoSorb®
CS: CytoSorb®
ICU: Intensive care unit
IQR: Interquartile range
Intra: Between the hemofilter and the CS filter
PD: Pharmacodynamics
PK: Pharmacokinetic
Post: After the CS filter
Pre: Pre-hemofilter
SQL: Structured Query Language
Sup: Supplement
TDM: Therapeutic drug monitoring
*V*_*d*_: Volume of distribution
