# Correction: Impact of hemoadsorption with CytoSorb^®^ on meropenem and piperacillin exposure in critically ill patients in a post-CKRT setup: a single-center, retrospective data analysis

**DOI:** 10.1186/s40635-025-00723-1

**Published:** 2025-03-03

**Authors:** Golschan Asgarpur, Franz Weber, Peggy Kiessling, Nilufar Akbari, Fabian Stroben, Bernadette Kleikamp, Charlotte Kloft, Sascha Treskatsch, Stefan Angermair

**Affiliations:** 1 Freie Universität Berlin and Humboldt-Universität Zu Berlin, Department of Anesthesiology and Intensive Care Medicine, Charité—Universitätsmedizin Berlin, Campus Benjamin Franklin, Berlin, Germany; 2 Department of Clinical Pharmacy and Biochemistry, Institute of Pharmacy, Freie Universitaet Berlin, Kelchstr. 31, 12169 Berlin, Germany; 3 Graduate Research Training Program PharMetrX, Freie Universitaet Berlin/Universität Potsdam, 12169 Berlin, Germany; 4 Labor Berlin—Charité Vivantes GmbH, 13353 Berlin, Germany; 5 Freie Universität Berlin and Humboldt-Universität Zu Berlin, Institute of Biometry and Clinical Epidemiology, Charité—Universitätsmedizin Berlin, Charitéplatz 1, 10117 Berlin, Germany

**Correction: Intensive Care Medicine Experimental (2025) 13:7** 10.1186/s40635-025-00716-0

Following publication of the original article [1], it came to the journal's attention that the first two authors had (incorrectly) not been marked as sharing co-first authorship. The article has since been corrected. The authors thank you for reading this erratum and apologize for any inconvenience caused.
