# Supplementary material for: Impact of hemoadsorption with CytoSorb® on meropenem and piperacillin exposure in critically ill patients in a post-CKRT setup: a single-center, retrospective data analysis
Source: Intensive Care Med Exp. 2025 Jan 18;13:7. doi: 10.1186/s40635-025-00716-0 (PMC11741966; doi:10.1186/s40635-025-00716-0)
Supplement: Supplementary file 1 — Additional file 1. [file 40635_2025_716_MOESM1_ESM.docx]

**Supplementary material**

${CL}_{dial}=Qb\times(C_{Pre}-C_{Intra})/C_{Pre}$ Eq. S1

${CL}_{cyto}=Qb\times(C_{Intra}-C_{Post})/C_{Intra}$ Eq. S2

${CL}_{comb}=Qb\times(C_{Pre}-C_{Post})/C_{Pre}$ Eq. S3

${Relative difference}_{dial,comb}=\left( \frac{{CL}_{dial}-{CL}_{comb}}{{CL}_{comb}} \right)$ Eq. S4

${Median relative difference}_{Pre,Intra}=Median\left( \frac{\left| C_{Pre}-C_{Intra} \right|}{C_{Pre}} \right)$ Eq. S5

${Median relative difference}_{Pre,Post}=Median\left( \frac{\left| C_{Pre}-C_{Post} \right|}{C_{Pre}} \right)$ Eq. S6

${Median relative difference}_{Intra,Post}= Median\left( \frac{\left| C_{Intra}-C_{Post} \right|}{C_{Pre}} \right)$ Eq. S7

| **Wilcoxon signed-rank test** | | | | |
| --- | --- | --- | --- | --- |
| **Drug** | **Comparison** | **Test statistic** | **p-value** | **Result** |
| **Meropenem** | Pre vs. Intra | V = 946 | 2.27E-13 | Significant difference |
|  | Pre vs. Post | V = 946 | 2.27E-13 | Significant difference |
|  | Intra vs. Post | V = 457.5 | 0.8563 | No significant difference |
| **Piperacillin** | Pre vs. Intra | V = 351 | 2.98E-08 | Significant difference |
|  | Pre vs. Post | V = 351 | 2.98E-08 | Significant difference |
|  | Intra vs Post | V = 237.5 | 0.1182 | No significant difference |

**Table S1:** Results of the Wilcoxon signed-rank tests for meropenem and piperacillin for comparisons between Pre vs. Intra, Pre vs. Post, and Post vs. Intra with test statistic V, p-values and the results indicating the significance of differences. ￼

| **Wilcoxon signed-rank test with average Pre-, Intra- and Post-values per patient** | | | | |
| --- | --- | --- | --- | --- |
| **Drug** | **Comparison** | **Test statistic** | **p value** | **Result** |
| **Meropenem** | Pre vs. Intra | V = 120 | 6.104e-05 | Significant difference |
|  | Pre vs. Post | V = 120 | 6.104e-05 | Significant difference |
|  | Intra vs. Post | V = 42 | 0.3303 | No significant difference |
| **Piperacillin** | Pre vs. Intra | V = 45 | 0.003906 | Significant difference |
|  | Pre vs. Post | V = 45 | 0.003906 | Significant difference |
|  | Intra vs Post | V = 35 | 0.1641 | No significant difference |

**Table S2:** Results of the Wilcoxon signed-rank tests for meropenem and piperacillin for comparisons between Pre vs. Intra, Pre vs. Post, and Post vs. Intra with test statistic V, p-values and the results indicating the significance of differences based on with average Pre-, Intra- and Post-values per patient.


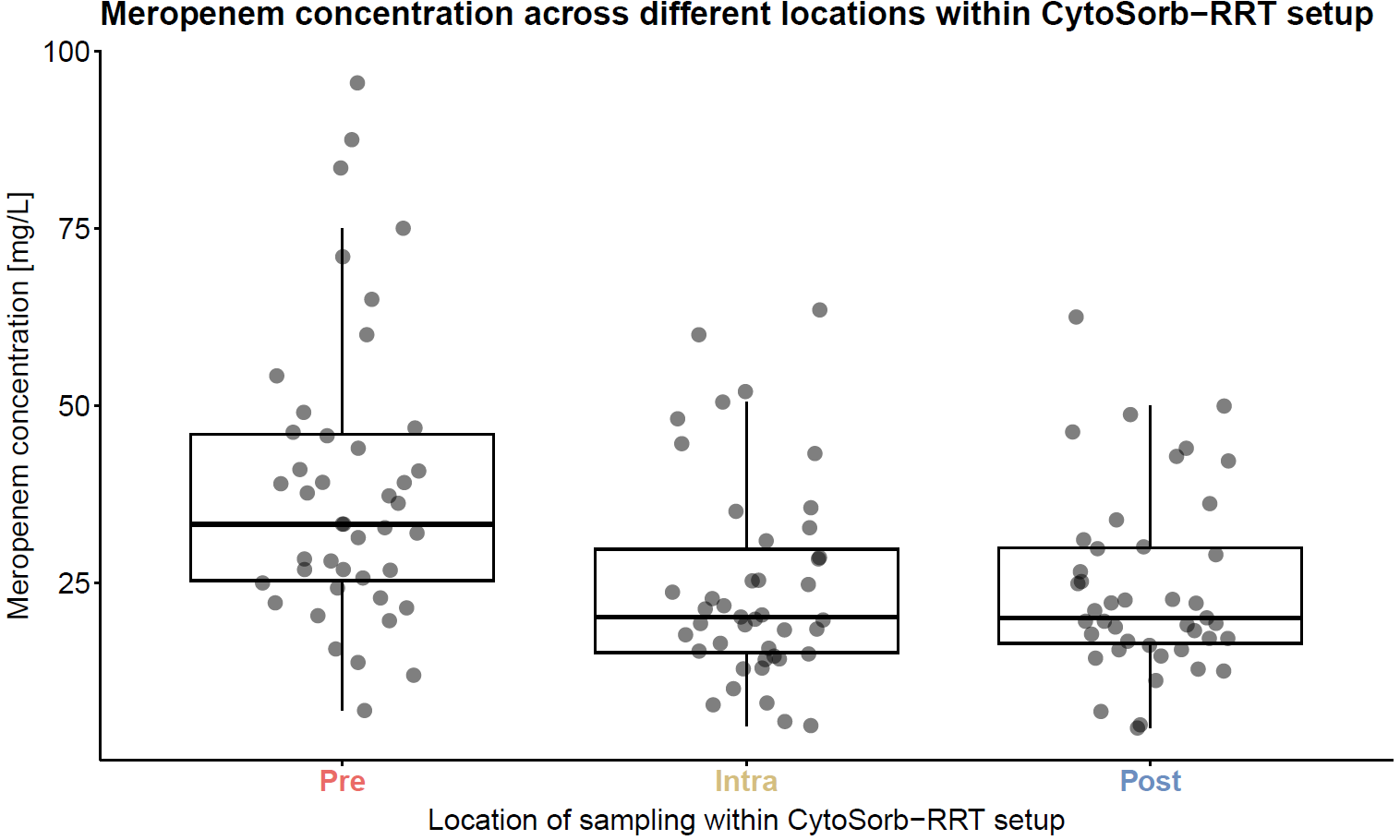


**Figure S1:** Meropenem concentration across different locations with CKRT-CS setup.


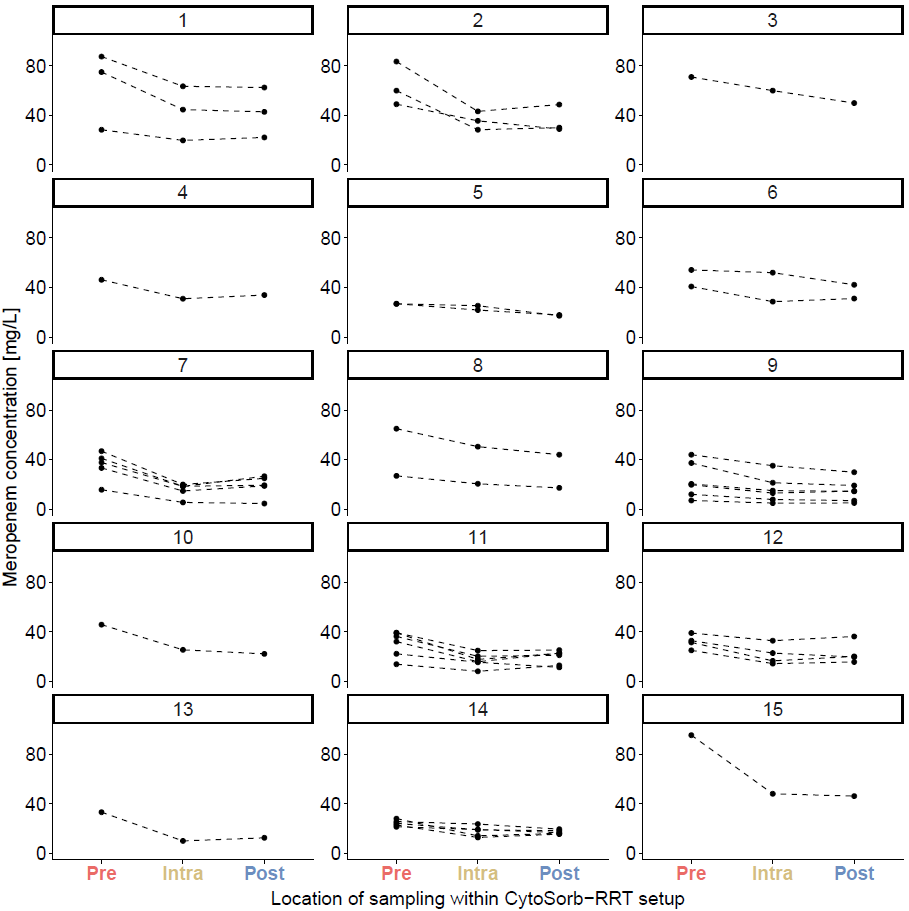


**Figure S2:** Meropenem concentration across different locations with CKRT-CS setup per patient.


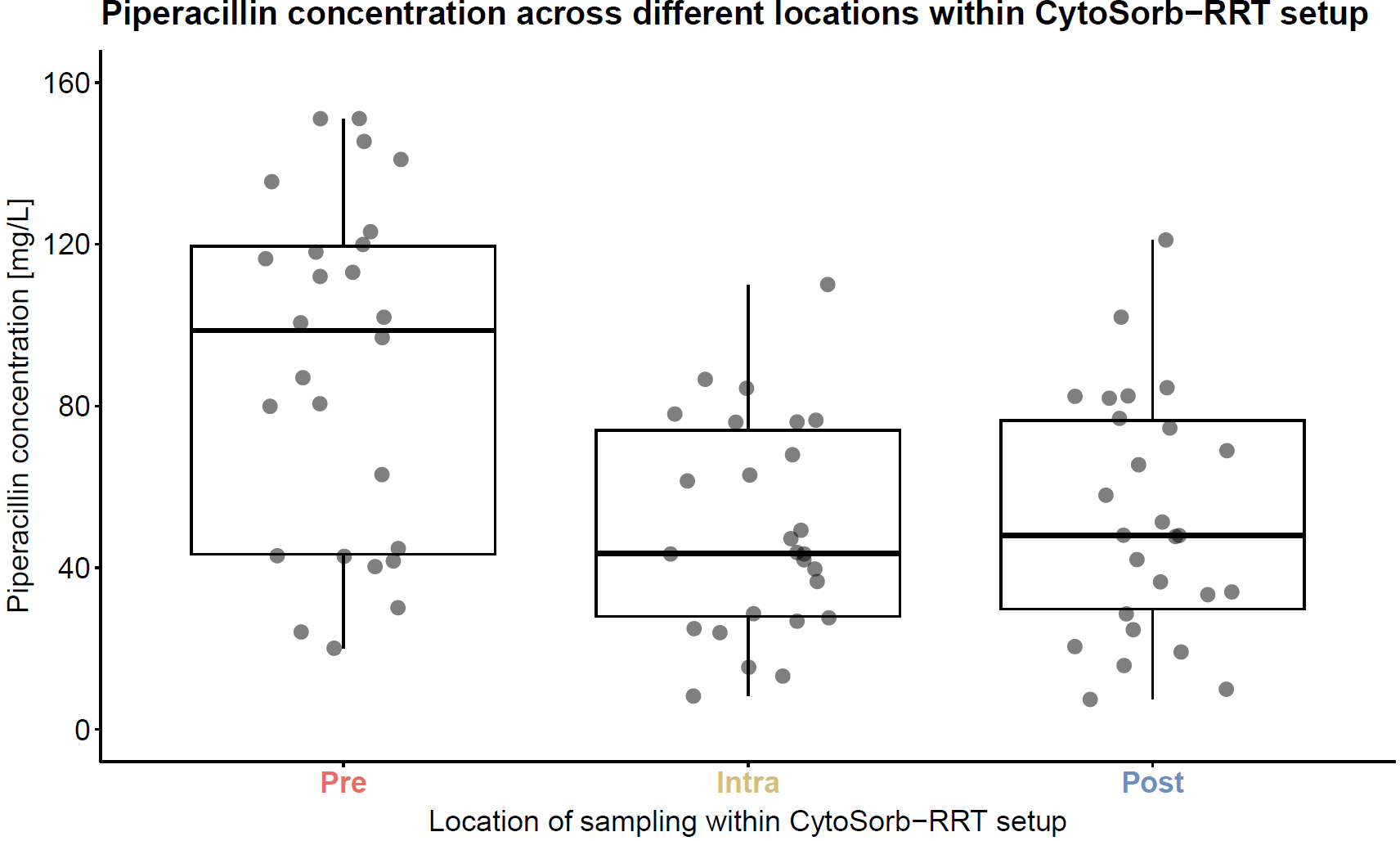


**Figure S3:** Piperacillin concentration across different locations with CKRT-CS set-up


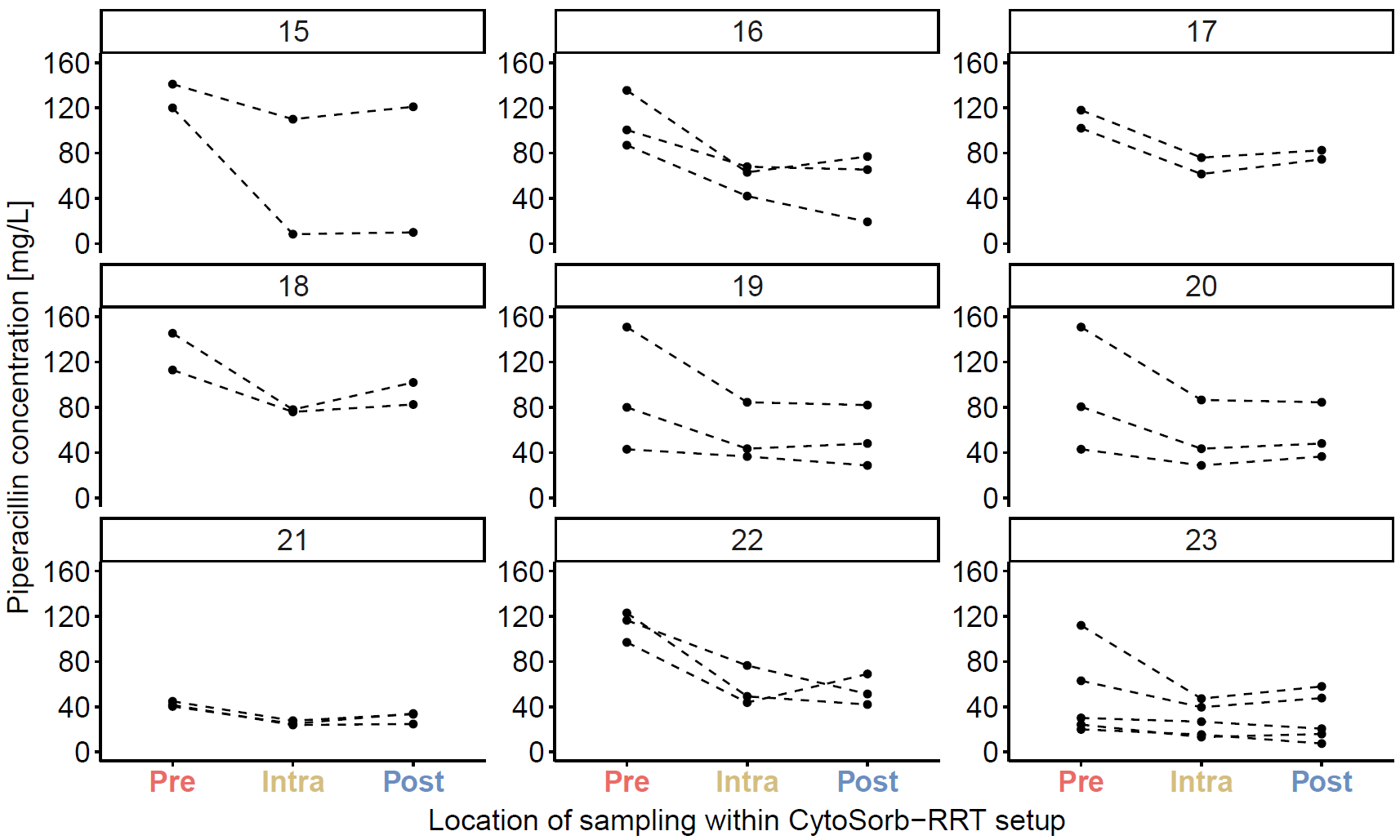


**Figure S4:** Piperacillin concentration across different locations with CKRT-CS set-up per patient


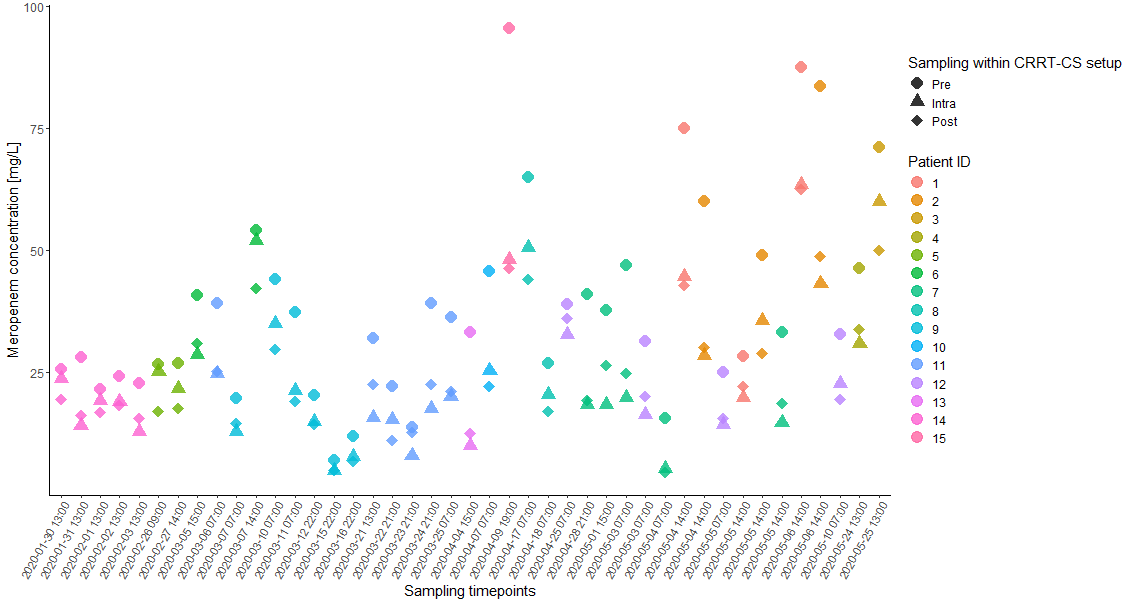


**Figure S5:** Meropenem concentration [mg/L] for different patients and sampling points within the study CKRT-CS setup. The plot illustrates the meropenem concentration at three different sampling points Pre, Intra, and Post taken simultaneously. Each point represents a sample collected from individual patients, with different shapes indicating the sampling location: Pre (circles), Intra (triangles), and Post (squares). The x-axis represents the specific time points of sampling, labelled chronologically. The colour of the points and lines corresponds to different patient IDs. ***Abbreviations:*** CKRT-CS setup: Continuous Renal Replacement Therapy followed by CS, Pre: before CKRT- CS setup, Intra: between CKRT and CS, Post: after CKRT-CS setup, ID: Identification number.


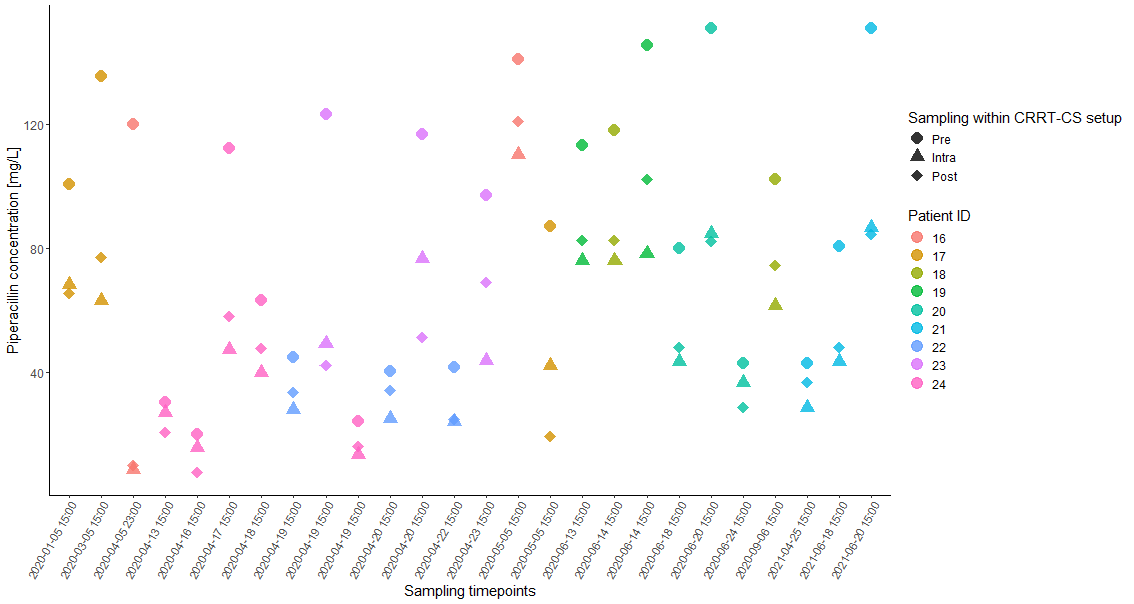


**Figure S6:** Piperacillin concentration [mg/L] for different patients and sampling points within the study CKRT-CS setup. The plot illustrates the piperacillin concentration at three different sampling points Pre, Intra, and Post taken simultaneously. Each point represents a sample collected from individual patients, with different shapes indicating the sampling location: Pre (circles), Intra (triangles), and Post (squares). The x-axis represents the specific time points of sampling, labelled chronologically. The colour of the points and lines corresponds to different patient IDs. ***Abbreviations:*** CKRT-CS setup: Continuous Renal Replacement Therapy followed by CS, Pre: before CKRT- CS setup, Intra: between CKRT and CS, Post: after CKRT-CS setup, ID: Identification number.
